# Impact of plant-based interventions on gynecological cancers: a narrative review of mechanistic interplays and clinical evidence

**DOI:** 10.3389/fmmed.2026.1832188

**Published:** 2026-06-23

**Authors:** Fidelis Batale Fabrael, John Sotunsa, Kehinde Samuel Olaniyi, Onome Bright Oghenetega, Oluwatobi Adewale, Blessing Monica Akindele, Faith Olanrewaju, Nekabari Lekpa Gbimadee, Seyi Olayemi, Abosede Oreoluwa Bolaji, Marvellous Osewe Iyobhebhe, Emmanuel Mololuwa Adegbotoluwa, Emmanuel Damilare Folahanmi, John Ahmadu Kaisar, Precious Adeoye Oyedokun

**Affiliations:** 1 Department of Biochemistry, Ahmadu Bello University, Zaria, Kaduna, Nigeria; 2 Reproductive Biology and Toxicology Research Laboratory, Oasis of Grace Hospital, Osogbo, Osun, Nigeria; 3 Department of Obstetrics and Gynecology, Benjamin Carson (SNR) College of Health and Medical Sciences, Babcock University, Ilisha Remo, Ogun, Nigeria; 4 Department of Physiology, Afe Babalola University, Ado-Ekiti, Nigeria; 5 Department of Physiology, Adeleke University, Ede, Osun, Nigeria; 6 Department of Biochemistry, Ladoke Akintola University of Technology, Ogbomoso, Oyo, Nigeria; 7 Axon Plus Research Consortium, Ogbomoso, Oyo, Nigeria; 8 Department of Physiology, Ladoke Akintola University of Technology, Ogbomoso, Oyo, Nigeria; 9 Department of Anatomy, Adeleke University, Ede, Osun, Nigeria

**Keywords:** apoptosis, bioavailability, cervical cancer, endometrial cancer, epigenetic regulation, gynecological cancers, HPV oncoproteins, nanoformulation

## Abstract

Gynecological cancers account for approximately 1.4 million new cases and 680,000 deaths annually. Cervical, ovarian, endometrial, vulvar, and vaginal cancers collectively impose a major and disproportionate burden on women in low- and middle-income countries. Conventional treatments, including surgery, platinum-based chemotherapy, and radiotherapy, are limited by systemic toxicity, acquired chemoresistance, recurrence, and financial inaccessibility in resource-limited settings. Approximately 80 percent of patients with advanced ovarian cancer eventually develop platinum resistance, and cervical cancer persists as a leading cause of cancer death in regions with inadequate screening and vaccination infrastructure. Plant-derived phytochemicals have attracted substantial scientific attention as adjunct or complementary anticancer strategies. This review synthesizes preclinical, epidemiological, and emerging clinical evidence for alkaloids, flavonoids, terpenoids, polyphenols, coumarins, and organosulfur compounds in gynecological cancer prevention and management. These compounds modulate multiple oncogenic pathways relevant to gynecological malignancies: tumor cell proliferation, intrinsic and extrinsic apoptosis, angiogenesis, oxidative stress, inflammation, hormone receptor signaling, HPV E6/E7 oncoprotein expression, and epigenetic regulation. Paclitaxel, a taxane terpenoid, serves as the definitive proof of concept that plant-derived compounds can achieve gold-standard clinical status in gynecological oncology. Critical barriers limit clinical translation for most non-approved phytochemicals: oral bioavailability is poor, botanical preparations are unstandardized, randomized trial data in gynecological cancer populations are scarce, and herb-drug interaction profiles are incompletely characterized. Nanoformulation approaches, including liposomal encapsulation, PLGA nanoparticles, and phytosomes, offer practical routes to overcome these pharmacokinetic obstacles. Future work should prioritize biomarker-embedded clinical trials, molecular subtype-stratified patient selection, and rigorous evaluation of phytochemical-chemotherapy combination regimens.

## 1 Introduction

Gynecological cancers refer to the cancer of the female reproductive system, such as ovarian, cervical, endometrial, vulvar, and vaginal cancers. These cancers collectively present a significant health burden across the world, with an estimated 1.4 million new cases and 680,000 deaths in 2020 alone (Liontos et al., 2022). Cervical cancer is the most common type of cancer, comprising 43.2% of all cases worldwide (Fowler et al., 2017). The disease burden caused by these cancers extends beyond physical burnout to individuals, caregivers, and communities (Liontos et al., 2022). Ovarian cancer is a form of cancer that arises in the ovaries and is often diagnosed in the late stages because of the subtle signs (Stewart et al., 2019). While cervical cancer originates from the cervix and is highly prevalent in developing countries due to limited access to HPV vaccination, inadequate screening programs (such as Pap smear testing), and delayed diagnosis and treatment of persistent infection with Human papillomavirus (Arbyn et al., 2020). Endometrial cancer is a type that is formed in the lining of the uterus and is usually common in developed countries due to higher rates of obesity, longer life expectancy, lower parity, and greater exposure to unopposed estrogen (Morice et al., 2016). Vulvar and vaginal cancers are rare types of gynecological cancers that develop in the external and internal parts of the female genital tract, specifically the vulva and the vagina (Crosbie et al., 2022). The incidence of gynecological cancers has greatly increased and has been projected to grow over the next 20 years (Eftekharzadeh et al., 2020). In Ukraine, uterine cancer has become more frequent than cervical cancer and demonstrates the difference in the types of cancers as well as mortality rates in different parts of the world (Ryzhov et al., 2020).

Conventional therapies for gynecological cancers, including surgery, chemotherapy, and radiation, face significant shortcomings that impact patient outcomes (Sarker, 2025). These challenges include toxicity, resistance, relapse, and high costs, which necessitate the exploration of alternative treatment strategies (Sarker, 2025). Traditional therapies cause severe multisystem toxicities which affect the quality of patient’s life (Brown and Chu, 2016). Chemotherapy, particularly agents such as cisplatin, may damage proliferating healthy cells, leading to long-term health problems (Brown et al., 2019). According to Lukanović et al. (2022), chemoresistance is a major challenge in chemotherapy, with approximately 80 percent of ovarian cancer patients developing resistance to platinum-based therapies (Lukanović et al., 2022). The mechanism of resistance involves disruption of the apoptotic pathway and increased drug efflux, making it harder to treat (Sharma et al., 2021). The recurrence of gynecological cancers is not uncommon, and most patients recover after treatment for the initial disease, which may be due to chemoresistance (Lukanović et al., 2022). And the financial cost of ongoing treatment for resistant cancers can be great, as these patients may require more expensive therapies or alternative treatments (Sharma et al., 2021).

Exploration of plant-based interventions for gynecological cancer treatment is driven by historical use, potential drug discovery from natural products, and accessibility. The traditional use of medicinal plants has been reported by many cultures for their therapeutic value; for example, in West Africa, more than 80% of people depend on medicinal plants for healthcare (Bayala et al., 2020). This traditional knowledge is a rich source from which researchers can assess the efficacy of these plant-based therapies against gynecological cancers. Traditional medicine used these remedies extensively to treat many ailments, including cancer. Many plants have also been reported to have medicinal applications for the treatment of gynecological problems; more than 60% of approved anticancer agents have plant origins, underscoring the importance of plant-derived materials in drug discovery (Bayala et al., 2020). Studies have now identified a wide range of plant-based compounds with anti-cancer activities, such as curcumin and epigallocatechin-3-gallate, that have shown efficacy against cervical cancer (Chakraborty, 2019). Plant-based remedies will potentially be easier to access and less costly for treating diseases, particularly in resource-strapped settings where conventional treatments may not be affordable (Chakraborty, 2019; Asiri et al., 2025). The utilization of such local flora for cancer cure can lead to better health outcomes in such communities and reduce damning economic and social burden of gynecological cancers.

This paper describes the potential benefits of plant-based interventions in the prevention and treatment of gynecological cancers. It synthesises evidence from different studies on the role of phytochemicals in patient’s outcomes and Quality of life. Phytochemicals have been found to have antitumor effects in cervical cancer, endometrial cancer, and ovarian cancer (Wozniak et al., 2021). Experimental studies have proven that these compounds have improvements in quality of life and cognitive function, and an inhibitory and synergistic effect compared to traditional therapies (Farrand et al., 2014). Epidemiological studies have suggested a correlation between plant-based diet consumption and a lower risk of cancer (Wozniak et al., 2021). A study conducted on women with metastatic breast cancer demonstrated that a whole food, plant-based dietary intervention produced very significant qualities of life and cognitive function improvement (Campbell et al., 2023).

Given the growing report of gynecological cancers in the world and the limitations posed with the conventional treatment options, there is growing interest to find safer options for the treatment as well as more effective and accessible treatment options. Plant-derived compounds have surfaced as potential candidates because of their diverse bioactive properties, relative potential to boost the effectiveness of therapy and relative low toxicity. Despite the growing body of evidence supporting the anticancer potential of these compounds, information on their role in gynecological cancer prevention and management remains fragmented. Therefore, this review aims to synthesise current evidence on plant-based interventions and phytochemicals with potential therapeutic relevance for gynecological cancers, highlighting their mechanisms of action, benefits, and future prospects for integration into cancer prevention and treatment strategies.

## 2 Methods and search strategy

### 2.1 Search strategy

We searched PubMed/MEDLINE, Scopus, Web of Science, and Google Scholar from database inception through May 2025, with priority given to publications from 2015 onward. Search terms were combined using Boolean AND/OR operators: (“plant-based compounds” OR “phytochemicals” OR “dietary polyphenols” OR “alkaloids” OR “flavonoids” OR “terpenoids” OR “organosulfur compounds” OR “coumarins”) AND (“gynecological cancers” OR “ovarian cancer” OR “cervical cancer” OR “endometrial cancer” OR “vulvar cancer” OR “vaginal cancer”) AND (“apoptosis” OR “cell cycle” OR “angiogenesis” OR “oxidative stress” OR “HPV oncogene” OR “epigenetics” OR “pharmacokinetics” OR “bioavailability” OR “clinical trial” OR “nanoformulation”). Vulvar and vaginal cancers were explicitly included in the search. The search was not restricted to polyphenols; all major phytochemical classes were included. The timeframe covered database inception through May 2025.

### 2.2 Inclusion and exclusion criteria

Studies were included if they were written in English, reported on plant-derived or phytochemical interventions in gynecological cancers, and addressed molecular mechanisms or clinical outcomes. Eligible study designs included randomized controlled trials, prospective and retrospective cohort studies, case-control studies, *in vitro* and *in vivo* experimental studies, and systematic reviews or meta-analyses. Studies were excluded if they were conference abstracts without peer-reviewed full text, were not available in English, or examined only non-gynecological cancers without mechanistic relevance to the gynecological context.

### 2.3 Data synthesis

Given the narrative design, no quantitative data pooling was performed. Where conflicting findings were identified, for example, discordant effects of phytoestrogens in different endometrial cancer cell line models, both results are presented and the methodological basis for the discordance is discussed. Evidence quality is evaluated contextually based on study design hierarchy, sample size, and methodological rigor.

### 2.4 Quality and risk of bias

Formal risk of bias tools (Cochrane RoB 2.0, SYRCLE for animal studies) were not applied in this narrative review. The methodological limitations of key cited studies are discussed in context throughout the text and in the Critical Analysis section. Most evidence for non-approved compounds derives from *in vitro* and animal experiments, and extrapolation to clinical settings requires caution.

## 3 Phytochemical classes and their activity in gynecological cancers

Phytochemicals active in gynecological cancer models fall into six structurally distinct classes: alkaloids, flavonoids, polyphenols, terpenoids, coumarins, and organosulfur compounds. The evidence below is organized by class, with mechanistic detail tied to named gynecological cancer cell lines and animal models. Table 1 provides a structured summary of 23 compounds across all classes.

**TABLE 1:** Summary of key phytochemicals, molecular targets, cancer types, and supporting evidence.

Phytochemical class	Key compounds	Cancer type	Molecular target/Pathway	Key findings	References
Alkaloid	Vincristine	Ovarian, cervical	Tubulin polymerization; microtubule disruption	G2/M arrest; induction of apoptosis in cisplatin-resistant ovarian cells	Lu et al. (2012)
Alkaloid	Camptothecin/Topotecan	Ovarian, cervical	Topoisomerase I inhibition; DNA strand breaks	Approved clinically for recurrent ovarian and cervical cancer; overcomes platinum resistance	Reyaz et al. (2025)
Alkaloid	Berberine	Endometrial, cervical	PI3K/AKT/mTOR; NF-κB suppression	Inhibits proliferation and induces autophagy in Ishikawa endometrial cells; sensitizes to cisplatin	Zhao et al. (2022)
Alkaloid	Colchicine	Ovarian	Microtubule depolymerization; spindle disruption	Anti-proliferative in SKOV-3 cells; synergizes with paclitaxel *in vitro*	Su et al. (2020)
Flavonoid	Quercetin	Ovarian, cervical	PI3K/AKT; Bcl-2/Bax; NF-κB; VEGF	Induces apoptosis and inhibits angiogenesis in HeLa and SKOV-3 cells; synergistic with cisplatin	Barboza et al. (2023)
Flavonoid	Genistein (isoflavone)	Endometrial, ovarian	ER-α/ER-β modulation; PI3K/AKT; CDK inhibition	Reduces endometrial adenocarcinoma incidence in mice; anti-proliferative at physiologic concentrations	Niwa et al. (2003)
Flavonoid	Apigenin	Cervical, ovarian	Caspase-3/9; PARP cleavage; Wnt/β-catenin	Induces apoptosis in HeLa and A2780 cells; suppresses HPV E6/E7 expression	Souza et al., 2017; Zhang et al., 2022; Asiri et al., 2025
Flavonoid	Luteolin	Endometrial, cervical	MAPK/ERK; HIF-1α; EMT inhibition	Blocks migration and invasion in endometrial and cervical cell lines; anti-angiogenic	Wang et al., 2018; Yadav et al., 2025
Flavonoid	Kaempferol	Cervical, ovarian, endometrial	STAT3; p53 restoration; caspase activation; JNK/ERK-CHOP pathway and upregulation of death receptors 4 and 5; PI3K/AKT	Promotes apoptosis in cisplatin-resistant ovarian cancer cells; reduces tumor volume in xenograft models; sensitizes human ovarian cancer cells-OVCAR-3 and SKOV-3 to tumor necrosis factor-related apoptosis-inducing ligand (TRAIL)-Induced apoptosis *via* JNK/ERK-CHOP pathway and upregulation of death receptors 4 and 5	Kashafi et al., 2017; Zhao et al., 2017
Polyphenol	Curcumin	Cervical, ovarian, endometrial	NF-κB; AP-1; COX-2; p53; E6/E7 suppression	Downregulates HPV oncogenes; sensitizes to cisplatin; clinical trials ongoing	Einbond et al., 2024; Munagala et al., 2011
Polyphenol	Resveratrol	Ovarian, endometrial, cervical	SIRT1; NF-κB; ERα; PI3K/AKT/mTOR	Induces apoptosis; inhibits metastasis; synergizes with paclitaxel in ovarian cancer	Mozafaryan et al. (2024)
Polyphenol	EGCG (green tea)	Cervical, ovarian	DNMT1 inhibition; HDAC; Wnt/β-catenin	Reactivates silenced TSGs *via* epigenetic reprogramming; phase II trial in cervical dysplasia	Ahn et al., 2003; Asiri et al., 2025; Singaravelan and Tollefsbol, 2025
Polyphenol	Cyanidin-3-O-glucoside (C3G)	Cervical; ovarian	ROS scavenging; Nrf2; Bax/Bcl-2	Reduces oxidative stress and induces apoptosis in granulosa tumor and ovarian adenocarcinoma cells	Zeng et al., 2012; Rugină et al., 2012; Liu et al., 2019; Behinska et al., 2025
Polyphenol	Naringenin	Cervical, endometrial	COX-2; 5-LOX; MAPK; caspase-3	Anti-inflammatory and pro-apoptotic in HeLa and Ishikawa cells at low micromolar concentrations	Zeng et al., 2014
Terpenoid	Paclitaxel (Taxol)	Ovarian, cervical, endometrial	Microtubule stabilization; mitotic arrest; Bcl-2 phosphorylation	FDA-approved; standard-of-care for advanced ovarian cancer; sensitizes *via* Bcl-2 downregulation	Qin et al. (2025)
Terpenoid	Artemisinin/Artesunate	Ovarian, endometrial	ROS generation; DNA damage; cell cycle arrest	Inhibits proliferation in cisplatin-resistant SKOV-3 cells; anti-angiogenic *via* VEGF suppression; induction of ROS; reduced proliferation; altered expression of cell cycle regulatory proteins, including cyclin D3, E2F-1 and p21; inhibition of mTOR signaling	Greenshields et al., 2017; Kamran et al., 2022
Terpenoid	Withaferin A	Cervical, ovarian	Proteasome inhibition; Hsp90; p53 restoration; E6/E7 suppression	Induces p53-dependent apoptosis; downregulates HPV18 oncoproteins in SiHa and HeLa cells	Munagala et al. (2011)
Terpenoid	β-Caryophyllene (BCP)	Ovarian	CB2 receptor; S-phase arrest; caspase-3/PARP	Induces caspase-dependent apoptosis in SKOV-3 ovarian cancer cells; anti-inflammatory	Arul et al. (2020)
Terpenoid	Betulinic acid	Cervical, ovarian	Mitochondrial permeability; cytochrome c; Bax/Bcl-2	Selective cytotoxicity toward cervical cancer cells over normal cells; induces intrinsic apoptosis	Xu et al., 2017; Kamran et al., 2022
Coumarin	Auraptene/Scopoletin	Ovarian, endometrial	PI3K/AKT/mTOR; MMP inhibition; multi-drug resistance reversal	Anti-invasive and anti-proliferative; reverses P-gp-mediated drug resistance in ovarian cancer cells	Jamialahmadi et al., 2018; Tian et al., 2019; Lin et al., 2025
Stilbene/Lignan	Tanshinone IIA	Cervical	HPV E6/E7 suppression; NF-κB; caspase-3	Synergizes with curcumin to inhibit W12 cervical precancer cell growth; targets integrated HPV	Einbond et al. (2024)
Organosulfur compound	Allicin (garlic-derived)	Ovarian, endometrial	ROS; Nrf2; Bax/Bcl-2; MAPK	Induces apoptosis and inhibits migration in ovarian cancer cells; antioxidant-mediated tumor suppression	Xu et al. (2014)

Abbreviations: PI3K, phosphoinositide 3-kinase; AKT, protein kinase B; mTOR, mammalian target of rapamycin; NF-κB, nuclear factor kappa B; VEGF, vascular endothelial growth factor; EMT, epithelial-mesenchymal transition; DNMT, DNA, methyltransferase; HDAC, histone deacetylase; HPV, human papillomavirus; TSG, tumor suppressor gene; ROS, reactive oxygen species; ER, estrogen receptor; MMP, matrix metalloproteinase; PARP, poly (ADP-ribose) polymerase; CDK, cyclin-dependent kinase. All studies cited support gynecological cancer-specific mechanistic evidence.

### 3.1 Alkaloids

Alkaloids, including vincristine and camptothecin, cause apoptosis, halt cell growth, and impair DNA replication pathways (Sharma et al., 2025). The use of alkaloids in traditional medicine has demonstrated their efficacy in treating various types of malignant diseases, thereby establishing their anticancer activity.

The application of alkaloids in traditional medicine has proven their efficacy in the treatment of many types of malignant diseases, thereby highlighting their anticancer activity. Alkaloids are among the most pharmacologically active groups of phytochemicals and have played an important role in the discovery of anticancer drugs (Lu et al., 2012). The fact that their application in gynaecological oncology is based not only on their biological activity but also on their development from natural products to clinically approved ERK-targeting therapeutics also informs us about the feasibility of plant-based compounds as a source of standardised cancer drugs (Reyaz et al., 2025). Many alkaloids have complex molecular structures, which provide them with a high potential to interact strongly with macromolecules in the cell, explaining their efficiency; however, this also requires caution when optimising their dose, focusing on possible toxicity (Heinrich et al., 2021). This duality highlights both the potential and the limitations of using alkaloids to treat cancer. In the context of plant-based interventions, a good example of this is the appearance of alkaloids, compounds that have crossed the boundary between traditional medicine and modern oncology, thus giving strength to the importance of ethnopharmacological knowledge in cancer therapeutic research.

Vincristine and vinblastine (Catharanthus roseus) arrest ovarian and cervical cancer cells in M phase through beta-tubulin binding, preventing microtubule polymerization. Their use in gynecological oncology combination regimens is established, though dose-limiting neurotoxicity restricts single-agent use (Reyaz et al., 2025). Topotecan, the semi-synthetic camptothecin derivative, inhibits topoisomerase I in replicating cells, generating DNA single-strand breaks that trigger apoptosis (Yakkala et al., 2023). Topotecan holds FDA approval for platinum-resistant recurrent ovarian cancer and advanced cervical cancer (GOG-179 trial), making it one of the most clinically relevant plant-derived drugs in gynecological practice.

Berberine, a protoberberine alkaloid from Berberis vulgaris, demonstrates consistent anti-proliferative activity in endometrial cancer models (Kuo et al., 2015; Liang et al., 2022). In endometrial carcinoma cells, including Ishikawa-derived models, berberine inhibits proliferation and promotes apoptosis through modulation of PI3K/AKT signaling, while broader cancer studies show concurrent AMPK activation and NF-κB inhibition (Kuo et al., 2015; Huang et al., 2021). Reported antiproliferative concentrations in related endometrial cancer models are commonly in the low micromolar range. 18beta-Glycyrrhetinic acid (GRA) from Glycyrrhiza glabra induces G0/G1 phase arrest through cyclin D1/CDK4 inhibition and activates caspase-3/9 in HPV18-positive HeLa cervical cancer cells, combining anti-proliferative and pro-apoptotic activity at the viral oncoprotein level.

### 3.2 Flavonoids

Flavonoids such as quercetin, genistein, and resveratrol modulate signaling pathways (e.g., NF-κB and MAPK), induce apoptosis, and inhibit angiogenesis (Sharma et al., 2025). Flavonoids are noted for their protective effects on healthy cells during chemotherapy (Li et al., 2019).

Flavonoids are a chemically diverse group of plant metabolites found in a wide range of food and medicinal plants and are thus of great importance in dietary and therapeutic interventions (Panche et al., 2016). Their significance in gynaecological cancer research is, in part, due to their frequent occurrence in hormone-responsive tissues and their accessibility over prolonged periods (Roy et al., 2022). In contrast to highly cytotoxic compounds, flavonoids have often been investigated for their more gradual effects on disease trajectories, thereby favouring their use in preventive, early, and supportive care. As they are widespread across plant-based diets, they strengthen epidemiological evidence of a link between nutrition and lower cancer risk (Halevas et al., 2022). In the field of plant-based cancer research, flavonoids are regarded as promising therapeutic agents with low toxicity, which can complement and potentially replace conventional treatments.

Quercetin (flavonol) induces mitochondrial membrane depolarization, cytochrome c release, and caspase-3/9 activation in SKOV-3 and A2780 ovarian cancer cells, accompanied by Bcl-2 downregulation. It suppresses VEGF transcription and NF-kB-driven proliferation in HeLa cervical cancer cells and demonstrates synergistic cytotoxicity with cisplatin in platinum-resistant ovarian cancer models, a mechanistically important observation for chemosensitization strategies (Barboza et al., 2023).

Apigenin (flavone) induces caspase-3/9-dependent apoptosis and PARP cleavage in HeLa cells and, distinctively, suppresses HPV E6/E7 oncoprotein expression in cervical cancer cell lines, targeting the viral driver of carcinogenesis rather than only the downstream oncogenic phenotype (Asiri et al., 2025). Kaempferol inhibits STAT3 phosphorylation and restores p53 function in cisplatin-resistant ovarian cancer cells, with tumor volume reduction demonstrated in A2780 xenograft models; STAT3 inhibition is mechanistically important because constitutive STAT3 activation is a well-characterized driver of platinum resistance in ovarian cancer (Wozniak et al., 2021). Luteolin suppresses HIF-1alpha and VEGF under hypoxic conditions, inhibits EMT, and reduces migration and invasion in Ishikawa endometrial and HeLa cervical cancer cell lines (Li et al., 2019).

Genistein and daidzein, soy-derived isoflavones, bind estrogen receptor beta with higher affinity than estrogen receptor alpha. In the endometrium, selective ER-beta agonism opposes ER-alpha-mediated proliferative signaling. Niwa et al. (2003) showed in mice that dietary genistein and daidzein significantly reduced the incidence of endometrial adenocarcinoma and atypical hyperplasia, and inhibited estrogen-induced oncogene expression. A meta-analysis of ten observational studies by Qu et al. (2014) reported a 49 percent reduction in ovarian cancer risk with higher phytoestrogen intake (RR 0.51, 95% CI 0.38–0.69). An important caveat is that the net estrogenic or anti-estrogenic effect of phytoestrogens depends on ambient estrogen levels, receptor subtype ratios, and cancer molecular subtype, producing conflicting results across different *in vitro* systems. This context-dependence must be acknowledged and should inform patient stratification in future trials.

### 3.3 Terpenoids

Terpenoids such as paclitaxel and artemisinin have anti-tumor, anti-metastasis, pro-apoptotic and anti-oxidative properties (Sharma et al., 2025). Terpenoids have been extensively used for medicinal purposes, both in ancient times and also in contemporary pharmacology. Terpenoids are one of the most structurally diverse class of plant phytochemicals, and include compounds that have well defined clinical relevance in the gynaecological treatment of cancer (Kamran et al., 2022). Their use is not restricted to its traditional applications and some terpenoids have been used in the conventional treatment of chemotherapy, especially in the treatment of ovarian and cervical cancer (Zhang et al., 2025). This class is biologically active and offers good pharmaceutical development opportunities; the latter are frequently applied as the structure for semi-synthetic derivatives with new biological activities and better efficiency and safety. However, the complexity of terpenoid structures could pose challenges for large-scale extraction and formulation. Although these are the drawbacks, terpenoids still draw attention to plant-based oncology for their proven clinical value and scarcity of integration between traditional medicine and evidence-based cancer treatment (Masyita et al., 2022).

Taxus brevifolia contains paclitaxel that stabilizes polymerized microtubules, arrests cells in G2/M phase, and phosphorylates Bcl-2 causing apoptosis (Sati et al., 2024). In combination with carboplatin, it is the standard of care used worldwide in advanced ovarian cancer with a reported 5-year overall survival of ∼45 percent (Stewart et al., 2019; Havasi et al., 2023). Therefore, the albumin-bound nanoparticle formulation (nab-paclitaxel or Abraxane) not only eliminates the hypersensitivity associated with Cremophor EL vehicle, but also shows that a plant-derived drug can be formulated into a nanoformulation that has an improved tolerability profile that can be FDA-approved (Cucinotto et al., 2013).

Withaferin A from W. somnifera inhibits the proteasome and disrupts the function of the Hsp90 chaperone, and directly downregulates HPV16/18 E6/E7 in SiHa and HeLa cervical cancer cells, causing p53 protein accumulation and inducing p53-dependent apoptosis with IC50 values of 1–5 μM (Munagala et al., 2011). Artesunate causes generation of ROS in iron-rich tumor cells, and it leads to oxidative DNA damage and growth inhibition of the cisplatin-resistant SKOV-3 ovarian cancer cells along with the suppression of VEGF (Wang et al., 2015). Beta-caryophyllene inhibits cell proliferation in SKOV-3 cells by arresting them in the S phase and by cleaving caspase-3 and PARP *via* CB2 cannabinoid receptor signaling (Arul et al., 2020). Hinokitiol interferes with the activity of cyclin D1/CDK4 in Ishikawa, HEC-1A, and KLE endometrial cancer cells, leading to ROS-induced G1 arrest and p53-mediated apoptosis (Chen et al., 2021). Betulinic acid selectively permeabilizes the tumor mitochondria, resulting in the activation of intrinsic apoptosis, in the models of ovarian and cervical cancer (Xu et al., 2017; Kamran et al., 2022).

### 3.4 Polyphenols

Polyphenols like curcumin and naringenin work on molecular pathways that participate in cancer progression (such as COX-2, 5-LOX). Increased polyphenols intake are linked to lower cancer risk.

The polyphenols are a large group of plant-based compounds and are probably best known for their anti-cancer effect. Population-based studies on the association of polyphenol-rich diets with lower incidence and better outcomes of gynecological cancers provide supporting evidence for their relevance in these types of cancer. Polyphenols are generally considered to have favorable safety profiles compared to the more potent forms of the cytotoxic phytochemicals, and may require chronic exposure *via* diet or supplements to achieve therapeutic effects (Singaravelan and Tollefsbol, 2025). These attributes make them particularly suitable for applications in risk-reduction, survivorship care and as supplements to standard therapy. Within the context of green interventions, polyphenols offer a nutritional and preventive dimension beyond the therapeutic effects of other classes of phytochemicals (Arab et al., 2025).

Curcumin (Curcuma longa) suppresses NF-kB, AP-1, and COX-2, shifts Bax/Bcl-2 ratios toward apoptosis, and downregulates HPV E6 and E7 in cervical cancer cell lines (Munagala et al., 2011). In combination with tanshinone IIA, curcumin inhibits HPV-positive W12 cervical precancer cells at sub-IC50 doses of each agent alone, targeting both episomally maintained and chromosomally integrated HPV (Einbond et al., 2024). Clinical studies of oral curcumin have demonstrated acceptable safety at doses up to 8 g/day and suggest potential activity against HPV-associated cervical lesions (Einbond et al., 2024).

Resveratrol activates SIRT1, suppresses NF-kB, and inhibits PI3K/AKT/mTOR signaling. In paclitaxel-resistant ovarian cancer cell lines, it restores taxane sensitivity through complementary Bcl-2 phosphorylation and mitotic checkpoint modulation (Mozafaryan et al., 2024). Findings confirmed resveratrol tolerability at 1 g twice daily and pharmacodynamic PI3K/AKT pathway modulation in ovarian tumor biopsies (Hedayati et al., 2025). EGCG (Camellia sinensis) inhibits DNMT1, DNMT3a, and DNMT3b, reactivating silenced tumor suppressor genes in ovarian and cervical cancer cells (Singaravelan and Tollefsbol, 2025). Ahn et al. (2003) showed 69 percent histologically confirmed CIN 2/3 regression with EGCG (topical and oral, 200 mg per day) *versus* 10 percent in placebo controls, one of the strongest positive clinical signals for any non-approved phytochemical in gynecological cancer prevention.

Cyanidin-3-O-glucoside activates Nrf2-driven transcription of superoxide dismutase, catalase, and glutathione peroxidase in ovarian cells while shifting Bax/Bcl-2 ratios to induce apoptosis in ovarian granulosa tumor and adenocarcinoma cells (Behinska et al., 2025). Naringenin inhibits COX-2 and 5-LOX and activates caspase-3 in HeLa and Ishikawa cells at low micromolar concentrations.

### 3.5 Coumarins

Coumarins are benzopyranone-based compounds present in numerous plant species with well-characterized anticancer properties in gynecological malignancies. Auraptene and scopoletin exert anti-invasive and anti-proliferative effects in ovarian and endometrial cancer cells through combined inhibition of PI3K/AKT/mTOR signaling and matrix metalloproteinase (MMP) activity (Jamialahmadi et al., 2018; Tian et al., 2019; Lin et al., 2025). Importantly, coumarins demonstrate the capacity to reverse P-glycoprotein-mediated multidrug resistance (a critical contributor to platinum and taxane resistance in ovarian cancer) through direct P-gp inhibition and reduced MDR gene expression (Tripathi and Misra, 2016). This positions coumarin derivatives as mechanistically relevant adjuncts to overcome a central barrier in gynecological cancer chemotherapy.

### 3.6 Organosulfur compounds

Allicin and related organosulfur compounds derived from Allium sativum (garlic) induce apoptosis in ovarian and endometrial cancer cells through ROS generation, Nrf2 pathway activation, and Bax/Bcl-2 ratio modulation (Xu et al., 2014). Anti-migratory and anti-invasive effects have been documented in ovarian cancer cell lines, with mechanistic contributions from suppression of MMP-2 and MMP-9 gelatinase activity. While clinical evidence for allicin in gynecological cancers remains limited to epidemiological associations, the mechanistic profile supports continued investigation (Crane et al., 2014).

## 4 Molecular mechanisms of action of plant-based interventions in gynecological cancers

### 4.1 Pro-apoptotic effect of plant-based interventions in gynecological cancers

Plant-based interventions for gynecological cancers employ bioactive compounds from various plants and exert antitumor effects through multiple molecular mechanisms (Lu et al., 2025). These effects have been attributed primarily to phytochemicals, including phenolic acids, flavonoids, terpenes, and alkaloids. Phytochemicals prevent cancer cell proliferation by interfering with cell proliferation. For instance, flavonoids and phenolic compounds have been found to cause cell cycle arrest in ovarian cancer cells (Barboza et al., 2023; Lu et al., 2025).

In the case of cervical cancer, plant-derived compounds have been shown to suppress tumor cell growth by targeting specific cell growth pathways involved in cell proliferation (He et al., 2021). Apoptosis or programmed cell death is one of the major mechanisms by which phytochemicals can exert their anticancer effects. Some of the compounds, such as saponins and alkaloids, can cause cell death (apoptosis) in cancer cells, thereby interfering with the growth and metastasis of cancer (Barboza et al., 2023). Natural products have been shown to activate apoptotic pathways, leading to the selective killing of cancer cells (He et al., 2021).

Pro-apoptotic and anti-proliferative properties in gynecological cancers appear to arise primarily through cell-cycle arrest and caspase activation (Chen et al., 2021). According to studies by Arul et al. (2020) 18beta-glycyrrhetinic acid (GRA) induced G0/G1 phase arrest, while beta-caryophyllene (BCP) induced S phase arrest in cervical and ovarian cancer cells, respectively. Hinokitiol targets cyclin D1 and CDK4, inhibiting their activity and disrupting the cell cycle in endometrial cancer (Chen et al., 2021). GRA activates caspase three and 9, suggesting activation of the intrinsic apoptotic pathway. BCP and diosmetin also induce caspase activation, with BCP activating caspase-3, and PARP cleavage was observed in ovarian cancer (Arul et al., 2020; Wang et al., 2019). Compounds such as GRA and hinokitiol regulate ROS, which plays a major role in the apoptosis of cancer cells (Chen et al., 2021).

**FIGURE 1:**
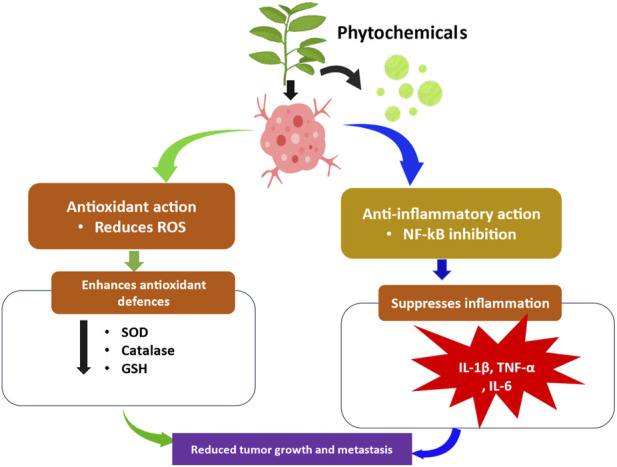
Pro-apoptotic and anti-proliferative effects of plant-based interventions in gynecological cancers. This figure depicts the suppression of gynecological cancer progression by plant derived phytochemicals such as phenolic acids, flavonoids, terpenes and alkaloids by inducing dual mechanisms. These compounds prevent the proliferation of tumor cells through arrest of the cell cycle (G0/G1 or G2/M) or by angiogenesis inhibition, and cell apoptosis by mitochondrial dysfunction, cytochrome-c release, caspase-3/9 activation, and increased Bax/Bcl-2 ratio. Together, these effects decrease tumor clonogenic survival, growth and metastasis.

Mechanistically, plant-based interventions targets dysfunctional balance between cell proliferation and programmed cell death that underlies gynecological cancers. At the cellular level, these effects are reflected in the alteration of cell cycle kinetics, decreased entry of S phase, G0/G1 or G2/M arrest, and mitotic arrest (Wang, 2021). Concurrently, there is induction of apoptotic signalling *via* both the intrinsic and extrinsic pathways through mitochondrial membrane depolarisation, cytochrome-c release, caspase-3 and caspase-9 activation and elevation of Bax/Bcl-2 ratios by phytochemicals (Rahman et al., 2021) (Figure 1). These coordinated changes lead to significant decreases in clonogenic survival, tumor cell viability and the proliferation indices (Ki-67) (Cidado et al., 2016). By simultaneously constraining cell-cycle progression and enhancing apoptotic competence, plant-derived compounds exert multi-layered control over tumor growth, a strategy that is particularly relevant in gynecological cancers with high proliferative indices and resistance to apoptosis (Chaudhry et al., 2022).

### 4.2 Antioxidant and anti-inflammatory pathways of plant-based interventions in gynecological cancers

Cyanidin-3-O-glucoside (C3G) found in berries C3G shows good antioxidant activity (protects ovarian cells from oxidative stress and induces apoptosis in cancer cells) (Behinska et al., 2025). Resveratrol has been shown to modulate several cellular processes, contributing to its anticancer effects in gynecological cancers (Mozafaryan et al., 2024).

**FIGURE 2:**
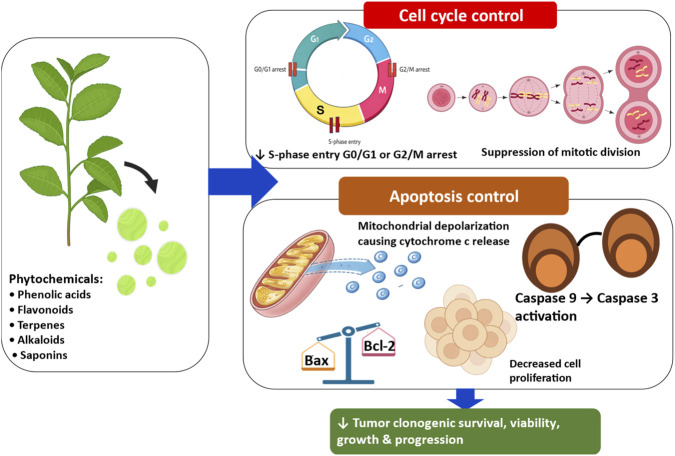
Plant-derived interventions modulate oxidative and inflammatory pathways in gynecological cancers. This diagram shows the antioxidant and anti-inflammatory effects of Cyanidin-3-O-glucoside (C3G) and resveratrol in inhibiting gynecological cancer progression. C3G decreases intracellular reactive oxygen species (ROS) and improves the endogenous antioxidant mechanisms, superoxide dismutase (SOD), catalase, and glutathione (GSH), restricting oxidative stress-established genomic instability. Resveratrol suppresses activation and nuclear translocation of NF-kB resulting in a decrease in expression of pro-inflammatory cytokines, such as IL-6, TNF-α, and IL-1β. These compounds target redox-sensitive and inflammation-mediated signaling in the tumor cells, which dampens proliferation, metastasis, and chemoresistance.

Polyphenols suppress NF-kB activation, which is associated with inflammation and the advancement of cancer (Figure 2). They inhibit the trans-location of NF-kB to the nucleus, which leads to less cell proliferation and metastasis (Khan et al., 2020). Natural extracts not only target NF-kB but also ROS, offering a dual-action mechanism that fights tumor growth and chemoresistance.

In parallel with their effects on tumor cell survival, plant-based interventions modulate oxidative and inflammatory pathways that sustain gynecological cancer progression (Carvalho, 2025). Elevated levels of reactive oxygen species (ROS), lipid peroxidation markers, and pro-inflammatory cytokines contribute to genomic instability and activation of oncogenic signaling (Yu et al., 2024). Phytochemicals counteract these processes by enhancing endogenous antioxidant defenses, including increased activity of superoxide dismutase, catalase, and glutathione-dependent enzymes, while reducing intracellular ROS accumulation (Cord et al., 2025). These biochemical changes disrupt inflammation-driven tumor support systems and attenuate redox-sensitive signaling cascades, thereby limiting the pro-tumorigenic microenvironment associated with gynecological malignancies (Chen et al., 2026).

### 4.3 Hormone receptor modulation of plant-based interventions in gynecological cancers

Hormone receptor modulation by phytoestrogens is a promising area for the development of hormone receptor-based interventions in gynecological cancers, especially endometrial and ovarian cancers. Phytoestrogens, plant-estrogen compounds, in which dietary consumption is cited to have estrogen-like activity, have been shown to affect several signaling pathways linked to these cancers, which could have preventive or therapeutic effects. Phytoestrogens, or plant-estrogen compounds, which are said to have estrogen-like activity, have been found to have various effects on signaling pathways associated with these cancers, with potential for both preventive and therapeutic applications (Kutuk and Kaplan, 2025). Phytoestrogens, such as soy isoflavones, may bind to the oestrogen receptors (ER-alpha, ER-beta) and the G-protein-coupled oestrogen receptor (GPER) to modulate cancer pathways (Kutuk and Kaplan, 2025). Qu et al. (2014) reported that higher phytoestrogen intake is associated with a decreased risk of ovarian cancer, especially food soy products, with a risk ratio of 0.51. Phytoestrogens could also disrupt steroidogenesis and downregulate protein tyrosine kinases, thereby exerting anti-cancer effects. Studies by Niwa et al. (2003) in mice have shown that genistein and daidzein significantly reduce the incidence of endometrial adenocarcinoma and atypical hyperplasia. These compounds inhibit estrogen-induced induction of oncogenes and inflammatory cytokines, suggesting a possible mechanism of their anti-aging effects (Niwa et al., 2003).

**FIGURE 3:**
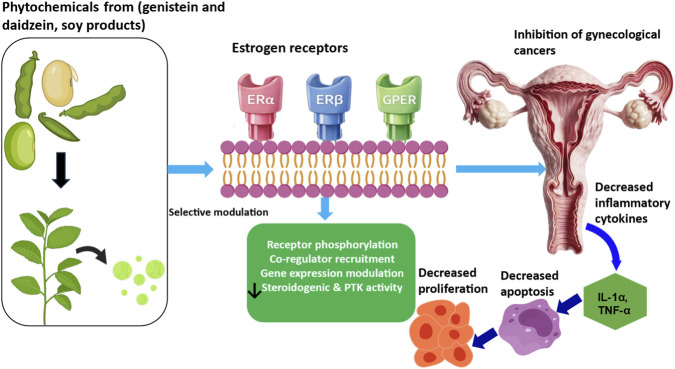
Phytoestrogen-mediated modulation of estrogen receptors and downstream signaling in gynecological cancers. This figure shows the interaction of phytoestrogens (including genistein, daidzein, and soy-derived compounds) with estrogen receptors at the cell membrane. Phytoestrogen binding causes selective modulation of receptors, manifested by changes in receptor activity and the activation of hormone-dependent signaling pathways. These downstream consequences include receptor phosphorylation, recruitment of co-regulators, estrogen response in gene expression, steroidogenesis, and protein tyrosine kinase signaling. The resultant cellular and tissue effects include estrogenic proliferation, enhanced apoptosis, inhibition of inflammatory cytokine and oncogene expression, and reduction of endometrial hyperplasia, which would, in aggregate, lead to a reduced risk and progression of hormone-dependent gynecological cancers, such as endometrial and ovarian cancers.

Hormone-dependent gynecological cancers exhibit aberrant estrogen and progesterone receptor signaling, which directly influences proliferation, apoptosis, and metabolic regulation (Chimento et al., 2022) (Figure 3). Plant-based interventions exert mechanistic effects through modulation of hormone receptor expression, receptor phosphorylation status, and downstream transcriptional activity (Cojocneanu Petric et al., 2015). These interactions can alter estrogen-responsive gene expression, reduce estrogen-driven proliferative signaling, and restore sensitivity to hormonal regulation (Cojocneanu Petric et al., 2015). Importantly, phytochemicals often function as selective modulators rather than full agonists or antagonists, producing tissue-specific effects that are reflected in altered receptor binding affinity, co-regulator recruitment, and transcriptional output. Such modulation provides a mechanistic basis for the observed influence of plant-derived compounds on hormone-responsive gynecological cancers while minimizing endocrine disruption (Figure 3).

### 4.4 Anti-angiogenic and anti-metastatic activity of plant-based intervention in gynecological cancer

Angiogenesis is necessary for tumor growth and metastasis. According to Barboza et al. (2023), phytochemicals block angiogenesis, thereby depriving the tumor of the nutrients and oxygen it needs to sustain itself. This mechanism is of particular relevance in the case of ovarian cancer, in which angiogenesis is involved in disease progression to a great extent (Lu et al., 2025). Various natural products, such as flavonoids and coumarins, have shown great efficacy in inhibiting angiogenesis and metastasis through different signaling pathways, especially by affecting the signaling pathways of vascular endothelial growth factor (VEGF) and the epithelial-mesenchymal transition (EMT). Flavonoids regulate vascular endothelial growth factor (VEGF) expression and block pathways such as NF-kB and PI-3K/Akt, thereby reducing angiogenesis and tumour growth (Subbaraj et al., 2021). Coumarins interfere with angiogenesis by inhibiting metalloproteinases and altering the PI3K/Akt/mTOR pathways, which are key to tumor cell migration and invasion (Karatoprak et al., 2024).

Amla (Emblica officinalis) extract has been shown to reduce SNAIL1 expression and increase E-Cadherin levels, and is an effective treatment for reversing EMT in resistant ovarian cancer cells (De et al., 2020). A review by Jia et al. (2024) identified 63 natural products that exhibit anti-angiogenic activity, highlighting their potential to modulate the tumor microenvironment and inhibit EMT.

**FIGURE 4:**
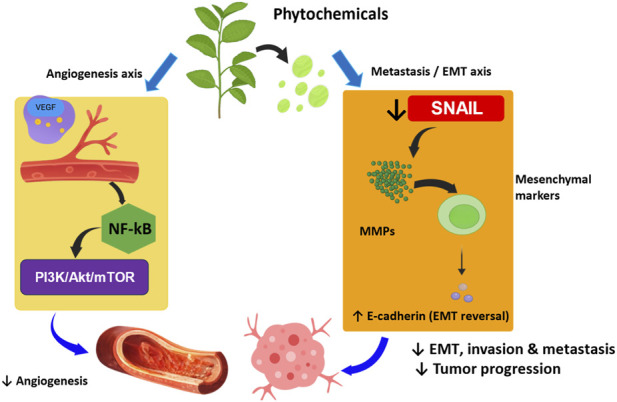
Anti-angiogenic and anti-metastatic mechanisms of plant-based interventions in gynecological cancers. The diagram depicts the anti-angiogenic and anti-metastatic mechanism through which plant-based compounds (flavonoids, coumarins, and amla extract) mediate protection against gynecological cancers. These agents inhibit angiogenic signaling induced by the growth factor vascular endothelial growth factor (VEGF) through the NF-kappa-beta (NF-kB) and phosphoinositide 3-kinase (PI3K)/Akt/mTOR pathways and modulate the epithelial-mesenchymal transition by downregulating the transcription factor SNAIL and MMPs, increasing epithelial cadherin (E-cad) expression. Together, these effects slow down the formation of blood vessels, tumor invasion, metastasis, and tumor progression.

Advanced gynecological cancers rely on angiogenesis and metastasis dissemination, which are governed by orchestrated molecular/cellular events (Yetkin-Arik et al., 2021). Plant-based interventions mechanistically disrupt these processes by suppressing the expression and function of angiogenic mediators, e.g., vascular endothelial growth factor and its receptors, and disrupt endothelial cell migration and tube formation (Hoseinkhani et al., 2020). At the metastatic stage, phytochemicals regulate epithelial-mesenchymal transition (EMT) proteins, reducing mesenchymal traits and restoring epithelial traits (Liu et al., 2023). This is accompanied by impaired matrix metalloproteinase activity, reduced extracellular matrix degradation, and decreased tumor cell motility and invasion (Liu et al., 2023). Collectively, these measurable changes reduce blood vessel support and metastatic potential, thereby limiting tumor and disease progression (Figure 4).

### 4.5 Targeting HPV oncogenes of plant-based interventions in gynecological cancers

The oncogenes E6 and E7, which play a key role in HPV-associated oncogenesis, can be effectively targeted by several phytochemicals that inhibit their expression and re-establish tumor suppressor pathways. Phytochemicals, such as Withaferin A and curcumin, have also been found to downregulate E6 and E7, resulting in apoptosis in cervical cancer cells by reactivating the p53 and other tumor suppressor proteins (Munagala et al., 2011) Combinations of plant compounds, such as curcumin and tanshinone IIA, have been found to enhance the inhibition of HPV oncogenes and promote cell death in cancerous cells (Einbond et al., 2024).

Plant-based therapies could have complementary treatments, which are less toxic and may have a great efficacy with HPV-related malignancies (Pal and Kundu, 2020). The use of phytochemicals may also be a preventive measure in HPV infection and further development of cancer (Einbond et al., 2024).

**FIGURE 5:**
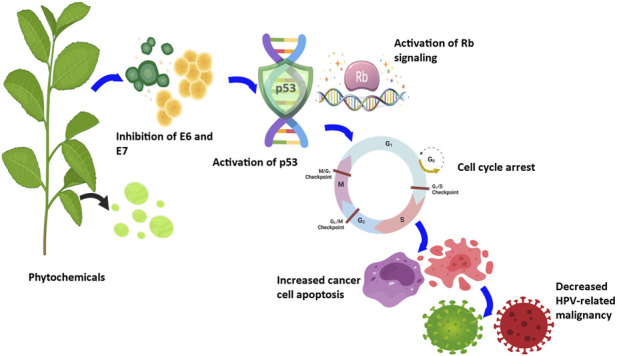
Phytochemical suppression of HPV oncogenes restores tumor suppressor signaling. The diagram illustrates the mechanism by which phytochemicals suppress carcinogenesis associated with HPV infection through action against the viral oncogenes E6 and E7, which are continuously highly expressed in HPV-associated gynecological cancers. Suppression of E6 and E7 prevents these proteins from inactivating the host tumor suppressors p53 and Rb, thereby reactivating these important regulatory processes. Restoration of p53 and Rb signals normalisation of cell cycle checkpoints and cell cycle arrest, induction of apoptotic cell death in HPV transformed cells. As a result, the rate of abnormal cell proliferation is weakened, and malignant cells are selectively destroyed; therefore, pharmacologically identifying phytochemicals as a mechanistically different and potentially less toxic method of treating HPV-associated malignancy.

In HPV-associated gynecological cancers, persistent expression of viral oncogenes E6 and E7 disrupts cell-cycle regulation and tumor-suppressor function (Pavelescu et al., 2025). Plant-based interventions target this virus-driven oncogenic axis by reducing viral oncogene expression and restoring host regulatory mechanisms (Gabbianelli et al., 2023). Mechanistically, this can be represented by reactivation of p53 and retinoblastoma protein signaling, normalization of cell cycle checkpoints, and reduction of virus-mediated genomic instability (Gabbianelli et al., 2023). These effects lead to decreased proliferation, increased apoptosis, and inability to transform affected cells. By explaining the viral component of carcinogenesis plus signaling abnormalities in the host, phytochemicals provide a unique mechanistic way of targeting HPV-induced gynecological malignancies (Figure 5).

### 4.6 Epigenetic modulation of plant-based interventions in gynecological cancers

Epigenetic modulation with plant-based interventions shows promising ways for the treatment of gynecological cancers, including ovarian cancer, endometrial cancer, and cervical cancer. These interventions primarily target alterations in DNA methylation, histone modifications, and microRNAs, which are critical epigenetic mechanisms that contribute to cancer progression (Bisht et al., 2024).

DNA methylation is one of the key events in carcinogenesis and frequently results in the silencing of tumor suppressor genes (Faa et al., 2016; Bisht et al., 2024). Aberrant methylation patterns can be observed by noninvasive liquid biopsies, which could be key to early diagnosis and monitoring of gynecological cancers (Bisht et al., 2024). Dietary habits, such as the intake of plant-based foods, can affect DNA methylation status, thereby lowering the risk of cancer (Faa et al., 2016).

Histone modifications, such as acetylation and methylation, play a dual role in gynecological cancers by either repressing tumor suppressor genes or activating oncogenes (Jacson and Mishra, 2020). These modifications play a major role in the formation and progression of cancers and can be used as biomarkers for early diagnosis and targeted therapy (Jacson and Mishra, 2020). Plant-derived compounds have demonstrated potential to modulate histone modifications and, consequently, affect the behavior of cancer cells (Chen et al., 2011).

MicroRNAs control gene expression by degrading messenger RNAs or inhibiting translation, influencing processes such as the cell cycle and apoptosis (Faa et al., 2016). The modulation of microRNAs by plant-based interventions may provide a new approach to cancer therapy by restoring normal gene expression patterns (Chen et al., 2011).

**FIGURE 6:**
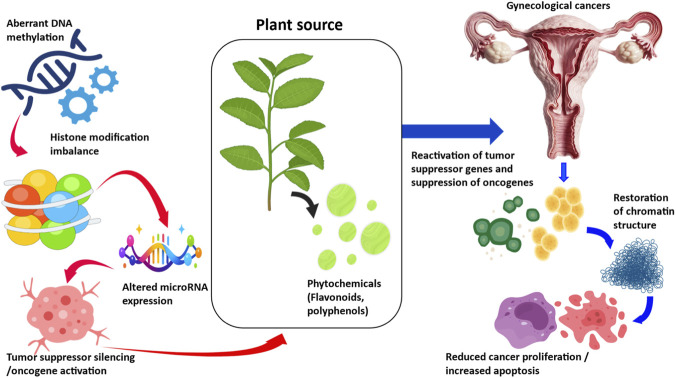
Phytochemical suppression of HPV oncogenes restores tumor suppressor signaling. Epigenetic disorganisation, such as enhanced DNMT activity, histone modification imbalance, and altered microRNA expression, has been found to drive the development of gynecological cancers, as these epigenetic changes can silence tumor suppressors and activate oncogenes. Plant-based bioactive compounds like polyphenols function as epigenetic materials by inhibition of DNMTs and HDACs and normalization of microRNA expression. This molecular correction restores chromatin structure, reactivates tumor suppressor genes, and inhibits oncogenes, thereby decreasing tumor growth rate and inducing apoptosis.

Epigenetic dysregulation contributes significantly to gynecological cancer development through aberrant DNA methylation, histone modification, and altered non-coding RNA expression (Marei, 2025). Plant-based interventions exert mechanistic effects by influencing epigenetic regulators such as DNA methyltransferases, histone acetyltransferases, and histone deacetylases (Akone et al., 2020). These interactions result in reactivation of silenced tumor suppressor genes and suppression of oncogene expression, leading to measurable changes in chromatin accessibility and transcriptional profiles. The reversibility of these epigenetic modifications aligns with the sustained, low-toxicity nature of plant-derived compounds and supports their role in long-term cancer management and prevention (Figure 6).

### 4.7 Synergy with standard therapies of plant-based interventions in gynecological cancers

Compounds such as curcumin, resveratrol, and genistein have been demonstrated to be strong chemosensitizers. They enhance responses to chemotherapeutic agents by blocking pathways that confer drug resistance, e.g., the PI3K/Akt/mTOR pathway, or by activating apoptosis in cancer cells (Garg et al., 2005; Karatoprak et al., 2024). Coumarins found in various plants have been shown to reduce multidrug resistance and enhance the efficacy of chemotherapy by inducing cell cycle arrest (Karatoprak et al., 2024).

Plant polyphenols, such as silymarin and green tea polyphenols, can sensitize tumor cells to combined radiation therapy by modulating oxidative stress and protecting against radiation-induced damage to normal cells (Garg et al., 2005). These compounds function by disrupting the pathways that lead to radiotherapy resistance and increasing the local effects of radiotherapy (Zhu et al., 2022; Garg et al., 2005).

Combining plant derivatives and conventional therapies have synergistic effects that result in better treatment outcomes with fewer side effects. Combination of the plant derivatives, along with the conventional therapies, brings about a synergistic effect resulting in the enhanced effectiveness of the treatment while bringing about fewer adverse effects. This approach is particularly useful to overcome the limitations of the currently employed treatments, including resistance and toxicity (Pezzani et al., 2019; Sharma et al., 2021).

The above mechanistic actions, when combined, augment the responsiveness of gynecological tumors to conventional therapies. Plant-based interventions modulate cell pathways involved in chemoresistance and radioresistance, including the DNA damage response, drug efflux transporters, and apoptotic signalling (Liu et al., 2024). These effects manifest as increased responsiveness of cancer cells to chemotherapy and radiotherapy, reduced toxicity in normal tissues at lower effective doses, and reduced overall toxicity. Mechanistically, this synergy stems from the phytochemicals’ ability to reprogram tumor biology at multiple regulatory levels, providing a strong rationale for their incorporation into standard gynecological cancer treatment regimens (Wozniak et al., 2021).

### 4.8 Combination effects with conventional therapy

Curcumin sensitizes platinum-resistant ovarian cancer cells to cisplatin by suppressing Bcl-2, survivin, and PI3K/AKT survival signaling, reducing the cisplatin dose required for equivalent cytotoxicity by 40–60 percent *in vitro*. Resveratrol reverses the paclitaxel resistance in ovarian cancer cell lines by acting in conjunction with the phosphorylation of Bcl-2 protein and the effect on the mitotic checkpoint (Mozafaryan et al., 2024). Genistein inhibits the activation of NF-kB in the radioresistant cervical cancer cells, thereby reducing the levels of anti-apoptotic proteins that make the radioresistant. Coumarin derivatives have been shown to inhibit P-gp expression, overcoming MDR in ovarian cancer models (Karatoprak et al., 2024). The ability of Silymarin and polyphenols of green tea to selectively spare normal tissue from radiation effects while leaving the tumor radiosensitive (Garg et al., 2005). The rationale for combination trials is clear: phytochemicals are programmed to act at several steps in the resistance process at the same time, which is very different from just adding cytotoxicity to an already established combination.

## 5 Clinical evidence and regulatory status

Mechanistic evidence on the phytochemical activity in gynecological cancers is extensive, whereas the clinical evidences are still underdeveloped (Table 2). Bringing the powerful preclinical results to a successful clinical trial is not without its significant pharmacological, regulatory, and trial design hurdles.

**TABLE 2:** Provides a comprehensive summary of key clinical and translational studies across phytochemical classes.

Phytochemical	Cancer type	Study type	Study phase/NCT number	Key outcome/Finding	Dosage used
Curcumin	Cervical cancer	Pilot clinical trial	NCT06080841	Investigating whether curcumin increases p53 expression and apoptosis in locally advanced cervical cancer during chemoradiotherapy	1 g, 3 g, or 6 g/day ± piperine
Curcumin + piperine	Cervical cancer	Pilot Interventional study	NCT06080841	Piperine added to improve curcumin bioavailability; safety and tumor apoptotic effects under evaluation	Curcumin 1–6 g/day with piperine
Curcumin	Cervical intraepithelial neoplasia (CIN)	Randomized phase II trial	NCI-2020-01951	Evaluating HPV clearance and regression of precancerous cervical lesions	Oral curcumin formulation (dose not specified in registry summary)
Curcumin (Curcugreen®)	Advanced cervical cancer (stage IIB)	Randomized placebo-controlled trial	NCT04294836 (Withdrawn)	Proposed to assess improvement in radiologic response, disease control, and survival with chemoradiation	500 mg every 6 h for 16 weeks
Nanocurcumin	High-risk HPV/Cervical cancer prevention	Randomized Double-blind clinical trial	Not specified	Improved HPV clearance compared with placebo (75% vs. 42.8%)	80 mg/day for 4 months
EGCG (green tea catechin)	Cervical dysplasia/HPV-related disease	Early clinical investigations	Various small clinical studies	Suggested anti-proliferative and HPV-suppressive effects; evidence still preliminary	Variable oral doses
Resveratrol	Ovarian cancer	Preclinical + translational studies	Early-stage exploratory studies	Demonstrated apoptosis induction and chemosensitization in ovarian cancer models	Variable experimental dosing
Genistein (soy isoflavone)	Ovarian and endometrial cancer	Observational/Translational studies	Early clinical evaluation	Potential modulation of estrogen signaling and tumor proliferation	Dietary or supplemental dosing varies
Quercetin	Ovarian cancer	Experimental/Translational studies	Preclinical with limited clinical translation	Demonstrated ROS-mediated apoptosis and synergy with chemotherapy	Not standardized
Curcumin	Ovarian cancer	Translational/Mechanistic studies	No large completed phase trials yet	May enhance platinum sensitivity and reduce chemotherapy resistance	Various nanoformulations under study

Paclitaxel and topotecan, both plant-derived, are FDA-approved for gynecological cancer indications and represent the benchmark for translational success from botanical oncology. Paclitaxel and carboplatin give about a 45 percent 5-year overall survival rate in advanced ovarian cancer. The approval of topotecan for platinum-resistant ovarian and advanced cervical cancer is based on data from Phase II/III trials, including the GOG-179 study.

Among non-approved phytochemicals, the strongest clinical signal is from EGCG in cervical precancer. The Phase II randomized trial of Ahn et al. (2003) showed that 69 percent of the patients with CIN 2/3 experienced histologically confirmed regression when taking oral and topical doses of 200 mg/day EGCG, compared to 10 percent of the placebo-treated patients. This is one of the last positive clinical controlled studies with a non-approved phytochemical for gynecological oncology. The results of the clinical trial (NCT06080841) indicate that curcumin up to 8 g/day is well tolerated and has been shown to result in a measurable reduction of the HPV E6/E7 oncoproteins in CIN lesions. The RR for intake of phytoestrogen and ovarian cancer risk reported by the meta-analysis by Qu et al. (2014) from 10 observational studies was 0.51 (95% CI: 0.38–0.69).

The other phytochemicals mentioned in this review such as withaferin A, berberine, artesunate and most flavonoids, coumarins, have no Phase II or III randomized trial data for gynecological cancer patients. There is a significant discrepancy between preclinical data and clinical results, which is the main limitation of this field. Future trials should include pharmacokinetic sampling, assessment of tumor markers, molecular subtyping and HPV status, and must utilize validated clinical endpoints, not just changes in tumor markers.

Key gap: The vast majority of phytochemicals with compelling preclinical evidence in gynecological cancer lack Phase II/III randomized clinical trial data. No phytochemical beyond paclitaxel and topotecan derivatives has achieved regulatory approval specifically for gynecological cancer indications. Future clinical trial design in this space must incorporate rigorous pharmacokinetic monitoring, tumor biomarker assessment, and patient stratification by molecular subtype.

## 6 Bioavailability, pharmacokinetics, and formulation

Poor oral bioavailability is the most significant obstacle to clinical translation for most non-approved phytochemicals in gynecological oncology. Curcumin has oral bioavailability below 1% in unformulated form due to rapid intestinal and hepatic phase II metabolism, primarily glucuronidation and sulfation (Aljabali et al., 2025). EGCG undergoes catechol-O-methyltransferase (COMT)-mediated methylation along with other phase II conjugation reactions, which substantially reduces its systemic stability and bioactive exposure (Aljabali et al., 2025). Resveratrol exhibits rapid first-pass metabolism, with reported plasma half-life of the parent compound ranging from minutes to low hours depending on formulation and analytical conditions (Aljabali et al., 2025). Quercetin bioavailability is highly variable across individuals and is influenced by gut microbiota composition, intestinal P-glycoprotein (P-gp) efflux activity, and food matrix–dependent absorption differences (Aljabali et al., 2025). Collectively, these pharmacokinetic constraints make it difficult to achieve tumor-relevant concentrations at tolerable oral doses, and *in vitro* anticancer effects observed at 10–50 µM cannot be directly extrapolated to clinical efficacy without bridging pharmacokinetic data from cancer patients.

Nanoformulation strategies directly address these limitations. Liposomal encapsulation improves aqueous solubility, protects phytochemicals from presystemic metabolism, and may enhance passive tumor accumulation *via* the enhanced permeability and retention (EPR) effect, although the magnitude of this effect in human tumors is variable. PLGA (poly-lactic-co-glycolic acid) biodegradable nanoparticles provide sustained and controlled drug release, thereby prolonging tumor-site exposure. Phytosomes, which are phospholipid–phytochemical complexes, enhance membrane permeability and have demonstrated improved systemic bioavailability for curcumin and resveratrol in human pharmacokinetic studies. Co-administration of piperine inhibits intestinal glucuronidation and modulates efflux transporters such as P-glycoprotein, resulting in approximately 20-fold increases in curcumin area under the curve (AUC) in human volunteer studies (Keyvani et al., 2024).

Nab-paclitaxel (Abraxane), derived from the plant-based chemotherapeutic agent paclitaxel, provides a clinically validated example of how nanoparticle formulation can improve the therapeutic index of a natural-product–derived anticancer drug. Its FDA approval and improved tolerability profile compared with conventional solvent-based paclitaxel establish an important regulatory and translational precedent for the nanoformulation of phytochemical-based therapeutics in oncology, including gynecological malignancies (Zhao et al., 2015). Table 3 summarizes key pharmacokinetic parameters and nanoformulation strategies for major phytochemicals with gynecological cancer relevance.

**TABLE 3:** Pharmacokinetic profiles and nanoformulation strategies for key phytochemicals.

Phytochemical	Oral bioavailability	Key PK limitation	Metabolic pathway	Nanoformulation/Strategy	Notes
Curcumin	Very low (<1% in unformulated human studies)	Poor solubility; rapid first-pass metabolism	UGT-mediated glucuronidation; sulfation (hepatic and intestinal)	Liposomes; PLGA nanoparticles; phytosomes; piperine co-administration	Piperine increases AUC by inhibiting glucuronidation and transporters
Resveratrol	Low (∼1% absolute; highly variable exposure)	Rapid clearance; short systemic persistence	UGT1A and SULT-mediated conjugation	Nano-encapsulation; cyclodextrin complexes; phytosomes	Parent compound half-life is short (minutes to low hours depending on study)
EGCG	Moderate (∼5–12%, variable)	Instability in neutral pH; rapid phase II metabolism	COMT methylation; glucuronidation; sulfation	Lipid nanoparticles; chitosan systems; pH-sensitive carriers	COMT metabolism reduces bioactive exposure
Quercetin	Variable (∼2–20% depending on form)	Efflux transport and microbiota-dependent metabolism	CYP1A2, CYP2C8; P-gp substrate; microbial deglycosylation	SNEDDS; phospholipid complexes; lipid carriers	Strong dependence on glycoside form and food matrix
Genistein	Moderate to high (food-dependent absorption)	Extensive conjugation; microbiota variability (equol production)	Intestinal β-glucosidases; CYP1A1/1B1; UGTs	Phytosomes; microencapsulation	More bioavailable than many polyphenols but rapidly conjugated
Paclitaxel	Negligible oral bioavailability	P-gp efflux; CYP3A4 metabolism; formulation toxicity issues (IV use)	CYP2C8; CYP3A4	Albumin-bound nanoparticles (nab-paclitaxel)	Clinically approved nanomedicine derived from natural product
Withaferin A	Low (poorly characterized)	Rapid clearance; high protein binding	CYP3A4; glucuronidation (preclinical evidence)	Polymeric nanoparticles (preclinical)	Human pharmacokinetics not well established
Berberine	Very low (∼5% or less)	P-gp efflux; intestinal metabolism	Gut wall CYP3A4; UGTs	Solid lipid nanoparticles; self-emulsifying systems	Extensive gut metabolism limits systemic exposure

## 7 Critical Analysis for reported evidence

### 7.1 Strengths and limitations of the evidence

The effective dose of plant-based therapies may be determined by the bioavailability of the active compound(s). For example, the effects of phytoestrogen absorption and metabolism, which are used to manage menopausal symptoms, may vary; thus, altering efficacy (Franco et al., 2016). Many phytochemicals have low aqueous solubility, rapid metabolism, and low systemic availability, which can result in a significant decrease in therapeutic concentrations at target tissues (Aljabali et al., 2025). Interindividual variability in gut microbiota composition, hepatic metabolism, and genetic polymorphisms further contributes to inconsistent pharmacokinetic profiles, complicating the translation of promising preclinical findings into predictable clinical outcomes.

In addition, the lack of standardization in preparations or dosages for plant-based remedies can lead to inconsistent therapeutic results. The heterogeneity in study quality and a high risk of bias in the clinical trials further complicate the studies of their efficacy (Franco et al., 2016; Abarnadevika et al., 2025). As such, variations in plant species, geographic origin, harvesting conditions, harvest methods, and formulation approaches often lead to substantial differences in the phytochemical composition reported in different studies. Variations in plant species, geographic origin, harvesting conditions, harvesting methods, and formulation approaches often lead to significant differences in the phytochemical composition reported across studies. Additionally, the generalizability of clinical investigations is limited by small sample sizes, short intervention durations, inadequate control groups, and the lack of reporting of randomization and blinding procedures, thereby reducing the strength and reproducibility of the conclusions drawn. In addition, the frequent substitutions made for endpoints that affect clinical outcomes limit the interpretability of the extant evidence.

Safety concerns exist due to side effects and interactions between the medications, plant-based therapies, and other medications. While traditional systems like Ayurveda and Siddha place greater emphasis on safety, participants in clinical trials are entitled to adverse events to verify some of these claims and ensure their safe use (Abarnadevika et al., 2025). Herb--drug interactions are still an understudied but clinically significant problem, especially in patients receiving chemotherapy or hormonal therapies for gynecological cancers. Certain phytochemicals may influence drug-metabolizing enzymes or transporters, potentially altering the efficacy or toxicity of conventional treatments (Gomez-Garduno et al., 2022). Moreover, the lack of long-term data means adverse effects may be overlooked due to the traditional view that natural products are inherently safe. This highlights the need for systematic pharmacovigilance, toxicity studies and efficacy studies. However, another one of the most significant drawbacks is the gap in the preclinical-clinical evidence. Plant-based compounds have demonstrated various beneficial properties against cancer and women’s health in many *in vitro* and animal studies, but these effects do not always translate to humans (Garcia-Oliveira et al., 2021). These differences in dosage, exposure time, and biological complexity between models and humans are responsible for this cross-species disconnect. In addition, synergistic effects seen under controlled laboratory conditions might not be achieved under clinical conditions without optimization of formulation/delivery systems. Although regulation and quality control issues are, for now, a challenge to getting plant-based therapies into mainstream healthcare. Herbal products are often marketed as a supplement, and not as a therapeutic product, so they are not as well regulated regarding their product quality, contamination or mislabeling (Dubale et al., 2025). This can cause variations in the quality of a product, contaminations or mislabeling, which affects the safety and efficacy of the product. To tackle these challenges, there needs to be a clear direction for standardization, a strong clinical trial design, and integration of traditional knowledge with evidence-based practice.

### 7.2 Herb-drug interactions and safety

Natural origin does not confer inherent safety, especially in oncology patients taking narrow-therapeutic index drugs. EGCG is an inhibitor of CYP3A4 and CYP2C9 enzymes that affect the metabolism of paclitaxel, cyclophosphamide and tamoxifen. Co-administration with piperine, which is commonly used to improve bioavailability of curcumin, is known to broadly inhibit P-gp as well as other CYP isoforms, and could significantly increase plasma levels of co-administered chemotherapy drugs. Genistein affects CYP1A1 and CYP1B1, which are involved in estrogen metabolism, and may influence the activity of the aromatase inhibitors (Gomez-Garduno et al., 2022). These interactions are clinically relevant and under-researched. Additionally, there is a special concern for phytoestrogen supplementation in the case of ER-positive endometrial (and ovarian) cancer: high-dose isoflavone supplementation in women taking hormonal therapy is not currently supported by the evidence and should be carefully evaluated as it could potentially reduce the effectiveness of such therapy.

### 7.3 Standardization and regulatory considerations

For applications of a botanical IND, FDA Botanical Drug Development Guidance states that consistency of composition, stability and manufacturing quality must be demonstrated. The majority of presently available phytochemical preparations are not fit for these requirements. The differences in plant species, geographic origin, harvest time and extraction process can lead to a wide range of curcuminoid content in a product that is marketed as curcumin extract. For the reproducibility of clinical trials and eventual regulatory approval, validated HPLC-MS/MS analytical fingerprinting is a prerequisite as well as Good Agricultural and Collection Practices throughout the supply chain and pharmacopoeial monographs that specify acceptable ranges (Wang et al., 2023).

## 8 Future research directions and clinical translation prospects

The results of critical synthesis of the existing evidence landscape result in several strategic research priorities. First, well-designed Phase II randomized controlled trials are needed to examine specific phytochemicals (or optimized nanoformulation thereof) in specific populations of gynecologic cancer patients (stratified by molecular subtype, HPV status, hormone receptor expression and prior treatments). To establish a full evidence base, biomarker-integrated trial design should include pharmacokinetic sampling, pharmacodynamic evaluation (e.g., circulating tumor DNA, tumor biopsy molecular profiling) and patient-reported outcome measures.

Secondly, the combination therapy paradigm should be studied systemically clinically. There is evidence of synergy in multiple preclinical studies, with phytochemicals and conventional cytotoxics, but no good clinical evidence of synergy exists. Combination IND should be designed using combination of phytochemicals and chemotherapies with appropriate safety run-in designs to determine tolerability prior to efficacy testing. Special attention should be paid to phytochemical combinations which target tumor cell-intrinsic resistance mechanisms (Bcl-2 overexpression, P-gp mediated efflux) and tumor microenvironment extrinsic mechanisms such as angiogenesis and immune exclusion.

Third, precision medicine strategies based on the genomomic and metabolomic profile of patients represent a promising pathway for the identification of patient subsets at greatest risk to respond to particular phytochemical interventions. For instance, patients with KEAP1/NRF2-mutant ovarian tumor type might be especially responsive to additional phytochemical-mediated oxidative stress dysregulators, while patients with high DNMT1 expression in cervical cancers might show preferential sensitivity to phytochemical-mediated epigenetic reprogramming. Implementing phytochemical therapy in the precision oncology framework is consistent with the wider paradigm of personalized medicine, and would greatly enhance the signal-to-noise ratio of clinical trials.

Finally, nanodelivery platforms warrant further development and clinical translation. Nanoparticles containing active targeting agents such as tumor specific ligands (folate receptor targeted nanoparticles for folate receptor over-expressing ovarian cancer, antibody conjugated nanocarriers for HER2 over-expressing endometrial cancer) would significantly increase therapeutic index. Considering the anatomical location of ovarian cancer (intraperitoneal cavity, which is accessible to regional therapy) the delivery strategy by thermosensitive liposomes is particularly interesting because of their ability to deliver phytochemicals to the area most affected by the disease spread.

Fifth, the specific evidence gap for vulvar and vaginal cancers (the rarest gynecological malignancies) requires targeted investigational attention. Targeted delivery of large concentrations of drugs at the site of the tumor, and low concentrations in the rest of the body, through a topical formulation of phytochemicals, may be especially effective for HPV associated vulvovaginal cancers, as was explored by Ahn et al. (2003) for HPV-associated cervical dysplasia. Investigated by Ahn et al. (2003) in cervical dysplasia. Exploratory trials with topical withaferin A, curcumin, or EGCG formulations in HPV-positive vulvar intraepithelial neoplasia would address a significant unmet need with mechanistically coherent and logistically feasible clinical designs.

## 9 Conclusion

This narrative review synthesizes current evidence demonstrating that plant-derived phytochemicals exert mechanistically diverse and potentially clinically relevant anticancer effects across the spectrum of gynecological malignancies. Across multiple phytochemical classes (alkaloids, flavonoids, polyphenols, terpenoids, coumarins, and organosulfur compounds) converging evidence from *in vitro*, *in vivo*, epidemiological, and emerging clinical studies supports the following mechanistic conclusions: (i) phytochemicals induce tumor cell apoptosis through both intrinsic (mitochondrial) and extrinsic (death receptor) pathways; (ii) they arrest cell cycle progression at G0/G1, S, and G2/M checkpoints through cyclin/CDK inhibition; (iii) they suppress angiogenesis and metastasis *via* VEGF pathway antagonism and EMT inhibition; (iv) they attenuate inflammatory and oxidative oncogenic microenvironments through NF-κB suppression and antioxidant enzyme upregulation; (v) they modulate hormone receptor signaling in hormone-sensitive endometrial and ovarian cancers; (vi) they target HPV E6/E7 oncoproteins in cervical cancer, restoring p53 and Rb tumor suppressor function; and (vii) they produce epigenetic reprogramming through DNMT and HDAC inhibition, reactivating silenced tumor suppressor gene networks.

The field is anchored by the clinical success of paclitaxel and topotecan derivatives (plant-derived compounds that have achieved gold-standard status in gynecological oncology) establishing the fundamental proof-of-concept that botanical compounds can yield transformative anticancer therapeutics. Emerging clinical signals from EGCG (69% CIN 2/3 response rate), curcumin (HPV oncoprotein suppression in Phase I/II), and resveratrol (pharmacodynamic target modulation in Phase I) support the translational potential of additional phytochemicals when developed with appropriate rigor.

However, critical limitations constrain current clinical translation: poor oral bioavailability for most non-approved compounds, absence of standardized preparations meeting regulatory specifications, limited high-quality randomized controlled trial evidence in gynecological cancer populations, and incompletely characterized herb-drug interaction profiles. Overcoming these barriers requires coordinated investment in nanoformulation development, botanical drug standardization infrastructure, biomarker-embedded clinical trial design, and pharmacovigilance systems appropriate for complex botanical products.

Future research should prioritize Phase II randomized trials of optimized phytochemical formulations in molecularly defined gynecological cancer patient populations, systematic investigation of phytochemical-chemotherapy combination regimens, and development of precision medicine frameworks to identify patients most likely to benefit from specific phytochemical interventions. Integration of plant-derived compounds into gynecological oncology (building on the transformative precedent of paclitaxel) represents a scientifically compelling and clinically urgent research priority.
